# Predicting Structural Motifs of Glycosaminoglycans using Cryogenic Infrared Spectroscopy and Random Forest

**DOI:** 10.1021/jacs.2c12762

**Published:** 2023-03-31

**Authors:** Jerome Riedel, Maike Lettow, Márkó Grabarics, Michael Götze, Rebecca L. Miller, Geert-Jan Boons, Gerard Meijer, Gert von Helden, Gergo Peter Szekeres, Kevin Pagel

**Affiliations:** † Department of Biology, Chemistry, and Pharmacy, Freie Universität Berlin, Berlin 14195, Germany; ‡ Department of Molecular Physics, Fritz-Haber-Institut der Max-Planck-Gesellschaft, 14195 Berlin, Germany; ¶ Department of Cellular and Molecular Medicine, University of Copenhagen, Copenhagen Center for Glycomics, Copenhagen N 2200, Denmark; § Bijvoet Center for Biomolecular Research, Utrecht University, 3584 CG Utrecht, The Netherlands; ∥ Complex Carbohydrate Research Center, University of Georgia, Athens, Georgia 30602-0002, United States

## Abstract

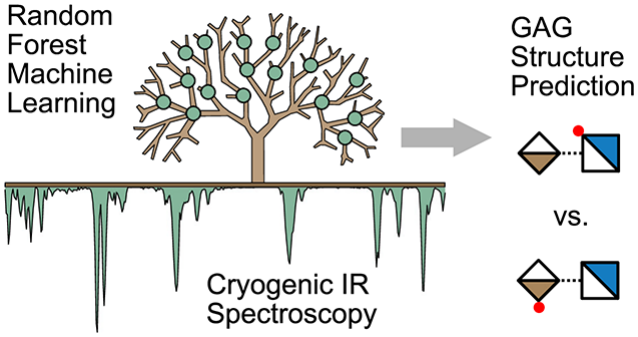

In recent years, glycosaminoglycans (GAGs) have emerged into the focus of biochemical and biomedical research due to their importance in a variety of physiological processes. These molecules show great diversity, which makes their analysis highly challenging. A promising tool for identifying the structural motifs and conformation of shorter GAG chains is cryogenic gas-phase infrared (IR) spectroscopy. In this work, the cryogenic gas-phase IR spectra of mass-selected heparan sulfate (HS) di-, tetra-, and hexasaccharide ions were recorded to extract vibrational features that are characteristic to structural motifs. The data were augmented with chondroitin sulfate (CS) disaccharide spectra to assemble a training library for random forest (RF) classifiers. These were used to discriminate between GAG classes (CS or HS) and different sulfate positions (2-*O*-, 4-*O*-, 6-*O*-, and *N*-sulfation). With optimized data preprocessing and RF modeling, a prediction accuracy of >97% was achieved for HS tetra- and hexasaccharides based on a training set of only 21 spectra. These results exemplify the importance of combining gas-phase cryogenic IR ion spectroscopy with machine learning to improve the future analytical workflow for GAG sequencing and that of other biomolecules, such as metabolites.

## Introduction

**Figure 1:**
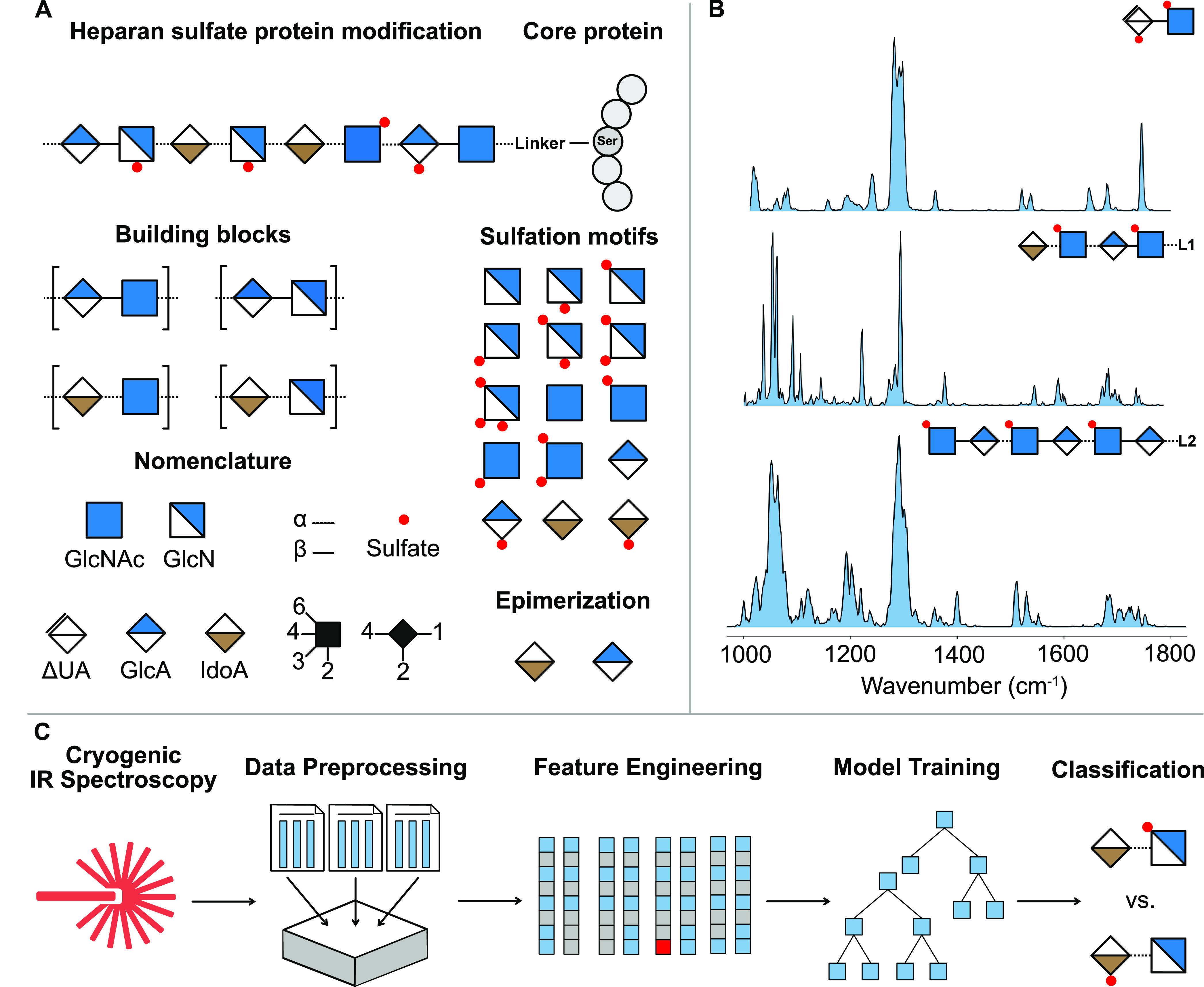
Random Forest analysis of glycosaminoglycan IR spectra. (a) Example of a heparan sulfate oligosaccharide linked to a protein. The structural complexity is defined by disaccharide building blocks, α/β-glycosidic bonds, and differences in sulfation degree and position.^31^ (b) Helium nanodroplet spectra capture the molecular fingerprint of GAGs. Structural motifs can be directly inferred from the position and intensity of vibrational bands in the spectrum as shown here for di-, tetra-, and hexasaccharides. (c) Schematic representation of the pipeline for training a Random Forest model starting from raw experimental data.

Glycosaminoglycans (GAGs) are linear sulfated polysaccharides that are involved in a variety of biological processes such as cell adhesion, blood coagulation, cell-to-cell communication, and regulatory interactions with chemokines and growth factors.^1,2^ Although GAGs consist of disaccharide units that are linked strictly linearly, they show great structural diversity arising from differences in chain length, monomer configuration, and sulfation degree and position. Generally, GAGs are divided into four major classes: heparin/heparan sulfate (Hp/HS), chondroitin sulfate/dermatan sulfate (CS/DS), keratan sulfate (KS), and hyaluronic acid (HA), with HS and CS exhibiting more diverse structures in comparison to KS and HA (Figure 1a).^3,4^ Sequencing GAGs is of paramount importance to understand their physiological activity and interactions; however, it remains challenging due to their structural complexity and the limited availability of pure samples.^5^ The overall composition of a GAG chain can be assessed using lyase digestion and subsequent liquid chromatography of the resulting disaccharides.^6^ Mass spectrometry-based experiments coupled with liquid chromatography, ion mobility, and different fragmentation techniques can further be used to identify characteristic oligosaccharide motifs,^4,7−9^ although the analysis of the fragmentation data is a complex task and may not always lead to unambiguous assignments.^4^

In the past few years, gas-phase vibrational spectroscopy of GAGs, and glycoconjugates in general, has emerged as a tool that provides complementary information to mass-spectrometry-based sequencing. Common gas-phase spectroscopic techniques range from infrared multiple photon dissociation experiments at ambient temperatures^10^ to tagging spectroscopy in cryogenic ion traps^11^ or helium nanodroplet spectroscopy.^12,13^ The obtained vibrational fingerprint can be correlated with the structural motifs and functional groups present within the molecule,^13−15^ which enables the identification and/or distinction of even minute structural details. Due to the ability of cryogenic gas-phase spectroscopy approaches to resolve even small shifts in band position, it has the potential to unravel the binding of GAGs to different target molecules. Here, changes in the IR spectrum of an intact and/or dissociated GAG complex might yield informative insights into GAG-specific interaction and binding patterns.

In this work, GAGs were studied using helium nanodroplet spectroscopy, where the ions are captured in superfluid helium nanodroplets and rapidly cooled down to 0.4 K. Irradiation with IR light leads to vibrational excitation of the ions in the droplet. The absorbed energy is redistributed from the ion to the matrix, resulting in partial evaporation and shrinking of the helium nanodroplets, thus maintaining a temperature of 0.4 K. Eventually, the trapped ion is ejected and can be detected in a time-of-flight mass spectrometer.^13,16^ Measuring molecules in a liquid-nitrogen-cooled ion trap that are picked up in helium nanodroplets at 0.4 K will purge the conformational ensemble from higher energy conformers. This results in a few low-energy conformers in their local minima, which effectively leads to decongestion of the IR spectrum.^17^ Moreover, the continuous cooling of ions by the superfluid helium will always relax the ions back to their vibrational ground state, thus reducing the spectral broadening. Therefore, ion spectra at cryogenic temperatures enable the systematic evaluation of vibrational band occurrences across a wide range of structural motifs.

Machine learning, especially cluster and pattern recognition algorithms are now frequently used in science to analyze complex data sets, as well as to solve problems where resource consumption is overwhelming.^18−20^ Random Forest (RF) modeling is among the more popular algorithms used for pattern recognition in IR and Raman spectroscopy, and has been successfully applied, e.g., to identify diseases in serum samples and to follow physiological processes in cells.^21−23^ Since its development by Breiman,^24^ RF became a well-established supervised machine learning technique and is known to perform well for medium to large size data sets, while having only minimal requirements on data type and feature correlation.^24−27^ RF classifiers train an ensemble of decision trees (CART algorithm) to partition the feature space into a network of decision rules that, upon traversal, answer the classification task. Given a data set with *n* samples and *k* features, RF models are trained on training sets holding *m* samples, where *m* ≤ *n*. A selection of features, which best partition the feature space, is directly incorporated in CARTs, but the selection can be suboptimal if the number of available samples is too low. In spectroscopic data, the dimensionality of the feature space regularly outweighs the number of samples available for training, which first requires feature reduction to avoid later misclassifications.^28^ In the past, different feature engineering strategies have been applied to reduce their number and to identify the most important features for the classification objective, e.g., principal component analysis, Wilcoxon testing, and evolutionary algorithms.^21,29,30^

Here, we present a workflow that establishes an IR spectral library of HS and CS di- and tetrasaccharides (Figure 1b) to train RF classifiers (Figure 1c) for predicting structural motifs in tetra- and hexasaccharide GAGs from their vibrational fingerprint. This approach opens up new possibilities to predict structural motifs in GAG oligosaccharides and helps with the identification of important GAG binding sequences for the design of novel pharmaceuticals. Moreover, it may serve as a blueprint for the analysis of other biomolecular classes, such as metabolites.

## Materials and Methods

A library of HS and CS disaccharide to hexasaccharide standards was assembled to cover the full chemical space of GAG features, including sulfation variations (*N*-sulfation versus *N*-acetylation, 2-*O*-, 4-*O*-, and 6-*O*-sulfation), epimerization (IdoA or GlcA) and backbone diversity (Hp/HS or CS/DS). The spectrum library includes 16 disaccharides (CS **1**–**8**, HS **9**–**16**), six tetrasaccharides (**17**–**22**), and one hexasaccharide (**23**) (Table S1). HS disaccharides were purchased from Iduron (Manchester, United Kingdom). All disaccharide standards have a reduced hexuronic acid (ΔUA) at the nonreducing end obtained by bacterial heparinase and -chondroitinase cleavage (β-elimination) of HS/CS oligosaccharides, respectively. The tetrasaccharides and hexasaccharide were synthesized chemically as described previously.^32,33^ Solvents (HPLC grade) were purchased from Sigma-Aldrich (StLouis, USA). Prior to use, all glycans were dissolved in water/methanol (v/v, 50/50%) to yield 50 μM analyte solutions. The gas-phase IR spectra for the library were recorded on the custom-built helium nanodroplet instrument in the range of 1000–1800 cm^–1^. A detailed description of the experimental setup can be found elsewhere.^13,16^

### Computational Details

The experimental IR spectra were extracted from time-of-flight intensity values measured as a function of the laser wavelength. The ion signal was then normalized to the repetition rate and energy of the laser as a first order approximation. Linear interpolation with 2 cm^–1^ steps was employed to align spectra on a commonly shared wavenumber axis before finding the largest shared wavenumber range among spectra (1010–1786 cm^–1^). In addition to the spectroscopic features, information about the charge and degree of sulfation was included, as well as on the presence of a linker when tetrasaccharides were part of the training set. The wavenumber features were binned by integration of the IR trace in windows of fixed width (15 cm^–1^, Figure S6) for the purpose of feature reduction (see Supporting Information, Data Pre-Processing). After spectral binning, bins were normalized to [0,1] to minimize the influence of absolute intensity values as latent variables in growing the decision trees, since in helium nanodroplet spectroscopy, intensities are not linear due to the absorption of multiple photons before the ions are ejected. This can result in over- or underestimating absorption cross sections of vibrational modes.^34^ For model training, feature selection was performed using an evolutionary optimization algorithm as implemented in the *GAFeatureSelectionCV* class provided by *sklearn-genetic-opt*.^35,36^ In each generation, the algorithm uses reproduction, mutation, and selection stages to choose the locally optimal features that will be present in the offspring generation. Construction of the offspring generation is made according to the μ + λ algorithm (see Supporting Information, Model Training).^37^ Additionally, elitism was used in each generation to maintain a set of the fittest features across evolutions. Classification was performed using an RF model trained by a Leave-One-Out cross-validation approach. Computationally, the *RandomForestClassifier* class provided by *scikit*_*learn* was used,^38^ which uses an optimized version of the CART algorithm for model training.^39^ The splitting criterion (partitioning of the sample space) at every node level was evaluated according to the entropy formulation. Detailed descriptions of the algorithms used for feature selection and model training can be found in the Supporting Information, along with a summary of the model training parameters used in the setup of feature selection and classifier classes (Tables S4 and S5 and Figures S6 and S7).

## Results and Discussion

**Figure 2:**
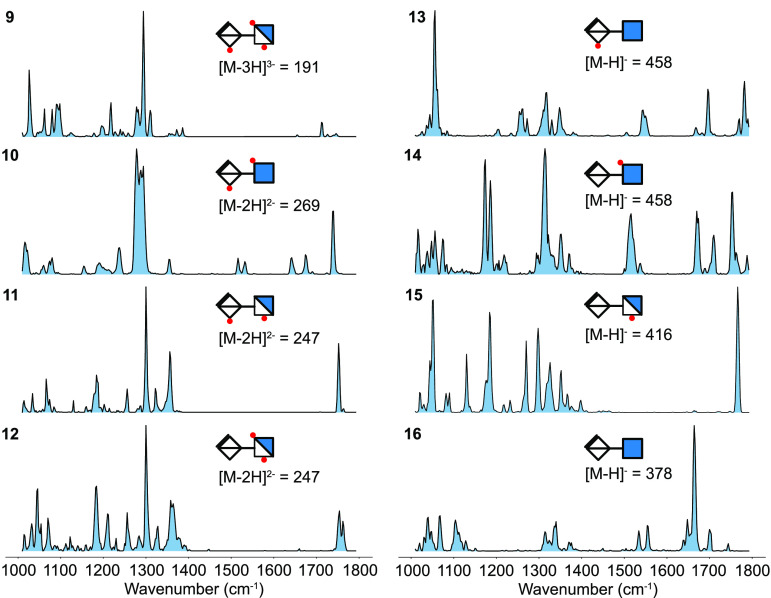
Helium nanodroplet spectra of HS disaccharides with varying sulfation patterns.

The set of HS di-, tetra-, and hexasaccharides form a diverse spectral library, from which prominent structural motifs can be inferred. The complete set of CS disaccharide and the **17**–**20** HS tetrasaccharide spectra were published previously (Figure S2 and S3).^14,15^ In Figure 2, the IR spectra of HS disaccharides **9**–**16** are shown in the range of 1000 to 1800 cm^–1^. Selected vibrational features (summarized in Table 1) were assigned based on previous experimental and theoretical results of glycan cations and anions.^14,40,41^ In sulfated GAGs, the 1000 to 1150 cm^–1^ range is populated by ν(C–O) and ν(C–C) modes, which overlap with the symmetric stretching of charged sulfates. The strong antisymmetric SO_3_^–^ stretching vibrations appear between 1150 to 1350 cm^–1^. These features serve as a unique fingerprint of the sulfation pattern in glycans. Further minor bands between 1200 and 1500 cm^–1^ can be assigned to C–H deformation and O–H bending modes. The distinct amide I–III modes between 1300 and 1700 cm^–1^ (of which amide III is generally very weak in IR spectra) stem from *N*-acetylation in GAGs. The amide I, with major contribution from ν(C=O), is typically the most intense amide region in the spectra, and it is highly sensitive to the intramolecular hydrogen bonding pattern associated with the conformation of the glycan.^15^ The amide II region is mainly populated by the N–H in-plane bending vibration with decreasing contributions from the C–N stretching motion and other minor components. The weak stretching mode ν(C=C) around 1600 cm^–1^ is characteristic for uronic acid monosaccharides derived from lyase digestion, which carry a C=C bond at the nonreducing end. Above 1700 cm^–1^, the C=O stretching vibration comes from the neutral carboxyl group in the hexuronic acid moiety. In nonsulfated GAGs, the charged carboxylate functional group yields a strong peak around 1700 cm^–1^, corresponding to the antisymmetric COO^–^ stretching.

**Table 1:** Tentative Assignment of Selected Vibrational Modes^a^ in Figure 2

Frequencies (cm^–1^)	**9**	**10**	**11**	**12**	**13**	**14**	**15**	**16**
ν_*a*_(SO_3_^–^)	1217, 1279, 1294, 1311	1238, 1278, 1287, 1294, 1355	1185, 1256, 1300, 1324, 1356	1183, 1212, 1316, 1283, 1301, 1327, 1361	1260, 1272, 1218, 1330, 1349	1173, 1186, 1218, 1296, 1314, 1323, 1334, 1351, 1370	1185, 1217, 1233, 1271, 1297, 1326, 1351, 1367, 1397	—
amide II	—	1517, 1533	—	—	1543, 1550	1515, 1538	—	1534, 1555
amide I	—	1641, 1675	—	—	1697	1670, 1710^b^	—	1647, 1701
ν(C=O)	1713	1739	—	1753, 1762	1769, 1782, 1790	1754, 1763, 1788	1766	—
ν_*a*_(COO^–^)	—	—	—	—	—	—	—	1664

a ν(ν_*a*_) designates the (antisymmetric) stretching mode.

b or ν(C=O) mode of the neutral carboxylic acid

All HS disaccharides **9**–**16** show unique IR signatures. *N*-acetylation (and therewith the lack of *N*-sulfation) is easy to determine due to the presence of the amide I (1600–1700 cm^–1^) and amide II (1500–1600 cm^–1^) vibrations in the IR fingerprints of disaccharides **10**, **13**, **14**, and **16**. A silent amide region can indicate that HS is either *N*-sulfated or has a free primary amine. This can later be circumvented by analyzing the 1294–1300 cm^–1^ range, where a single strong mode at ≈1300 cm^–1^ (or red-shifted in case of high sulfation) can indicate *N*-sulfation. The *O*-sulfation motif in these ions (2-*O*- or 6-*O*-sulfation) is more challenging to differentiate based only on the IR spectra.

**Figure 3:**
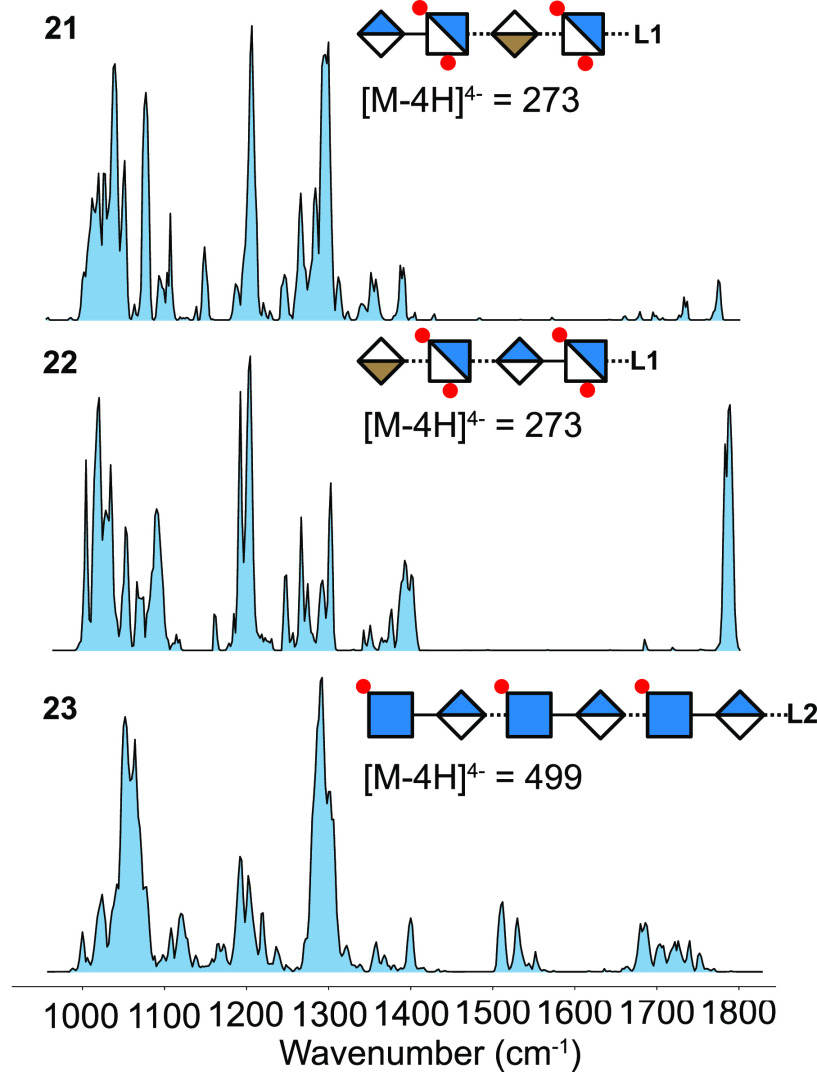
Helium nanodroplet spectra of HS tetrasaccharides and hexasaccharide from 1000 to 1800 cm^–1^. The tetrasaccharide diastereomers were labeled with an aminopentyl linker (L1), while the hexasaccharide carried a *p*-methoxyphenyl linker (L2).

The number of conformers captured in the cryogenic ion trap can vary even for ions of very similar chemical structure.^15^ A double band in the IR spectrum of disaccharide **10** with a shift in the amide I band of ≈35 cm^–1^ can indicate that two conformers (or anomers) were present in the trap. The number of conformers in the cryogenic ion trap for disaccharides **9**, **11**, and **15** appeared to be rather low because the neutral carboxylic acid only yields a single ν(C=O) mode. In the sulfated HS disaccharides, this band shows an apparent tendency: with increasing sulfation, the ν(C=O) stretching vibration red-shifts from the 1753–1781 cm^–1^ region (singly sulfated) to the 1739–1762 cm^–1^ region in the doubly sulfated HS ions, and eventually to 1712 cm^–1^ in the triply sulfated HS ion. This red-shift is most probably a contribution of the electron-withdrawing characteristics of sulfates, with increasing sulfation reducing the electron density around the carboxyl moiety. In the spectra of **11**, **12**, and **14**, the ν(C=O) band appears at 1753 and 1763 cm^–1^ as a double peak, while the region below 1400 cm^–1^ is more populated with (often weak) modes compared to the other spectra. This leads to two conclusions: these three ions can exist in a more extended conformational space than the other ions, while they also share a highly similar chemical environment around the carboxyl moiety. The ν(C=O) region in the spectrum of **13** contains several additional bands, with unique band positions compared to the previous case pointing toward a different chemical environment for the carboxylic moiety. This can result from the fact that the only charged sulfate in this case is located on the same monosaccharide unit as the carboxyl group itself. The three charged sulfates in disaccharide **9** show intense ν_*a*_(SO_3_^–^) vibrational features in the IR spectrum, which can overpower the ν(C=O) and account for its relatively low intensity. It is important to note that the relative intensities only serve as a qualitative measure in helium nanodroplet spectroscopy.^34^ Interestingly, the most intense band in the IR spectrum of disaccharide **13**, which carries 2-*O*-sulfation, is at 1055 cm^–1^. The ν(C–O) and ν(C–C) of the glycan core and the ν_*s*_(SO_3_^–^) in this region are difficult to differentiate; therefore, this strong mode remains unassigned. The cryogenic IR spectra of the HS tetrasaccharides **21** and **22** are shown in Figure 3. The two diastereomers can be differentiated from their IR signature (Table 2). The aminopentyl linker at the reducing end has additional contributions in the 1200–1500 cm^–1^ region corresponding to C–H deformation, while the scissoring motion of neutral, primary amines are typically found around 1600 cm^–1^ and are of very low intensity (Table 2).^42,43^ This highly flexible linker can form interactions of different strength with several different functional groups in the molecules, which may distribute their vibrational contribution over an extended wavenumber range, thus resulting in an overall weak NH_2_ signal.

**Table 2:** Tentative Assignment of Selected Diagnostic Modes^a^ in the Spectra of HS Tetra- and Hexasaccharides 21–23^14^

Frequencies (cm^–1^)	**21**	**22**	**23**
ν_*a*_(SO_3_^–^)	1150, 1207, 1267, 1286, 1296, 1301, 1357, 1393	1160, 1191, 1203, 1247, 1266, 1274, 1292, 1302, 1392, 1400	1192, 1202, 1290, 1301, 1400^b^
amide II	—	—	1511, 1530
amide I	—	—	1684, 1704^b^
ν(C=O)	1739, 1780	1781, 1787	1725, 1740

a ν(ν_*a*_) designates the (antisymmetric) stretching mode.

b or ν(C=O) mode of the neutral carboxylic acid

### Random Forest

Since the data set of di-, tetra-, and hexasaccharide samples is small compared to those usually used in RF model training, one of the main tasks was to optimize the feature-to-sample ratio. Otherwise, RF models are prone to overfitting, which in the case of IR spectra results in the selection of vibrational features that are not associated with the structural motif in question. For classification models working on high correlation data sets, such as IR spectra, in which neighboring IR channels are highly correlated, the optimal feature-to-sample ratio can be approximated by  features selected in the final model.^28,44^ Furthermore, at every node level during training of the decision trees in the RF model, a set of  features is considered for partitioning, where *k* is the total number of features in the training set.^44,45^ With a total number of 59 features after spectral binning, additional feature selection by an evolutionary algorithm had to take place to constrain the size of the feature space and mitigate overfitting effects. This step resulted in seven features used per decision function on average, with a maximum of 23 features used for HS/CS classifications.

**Figure 4:**
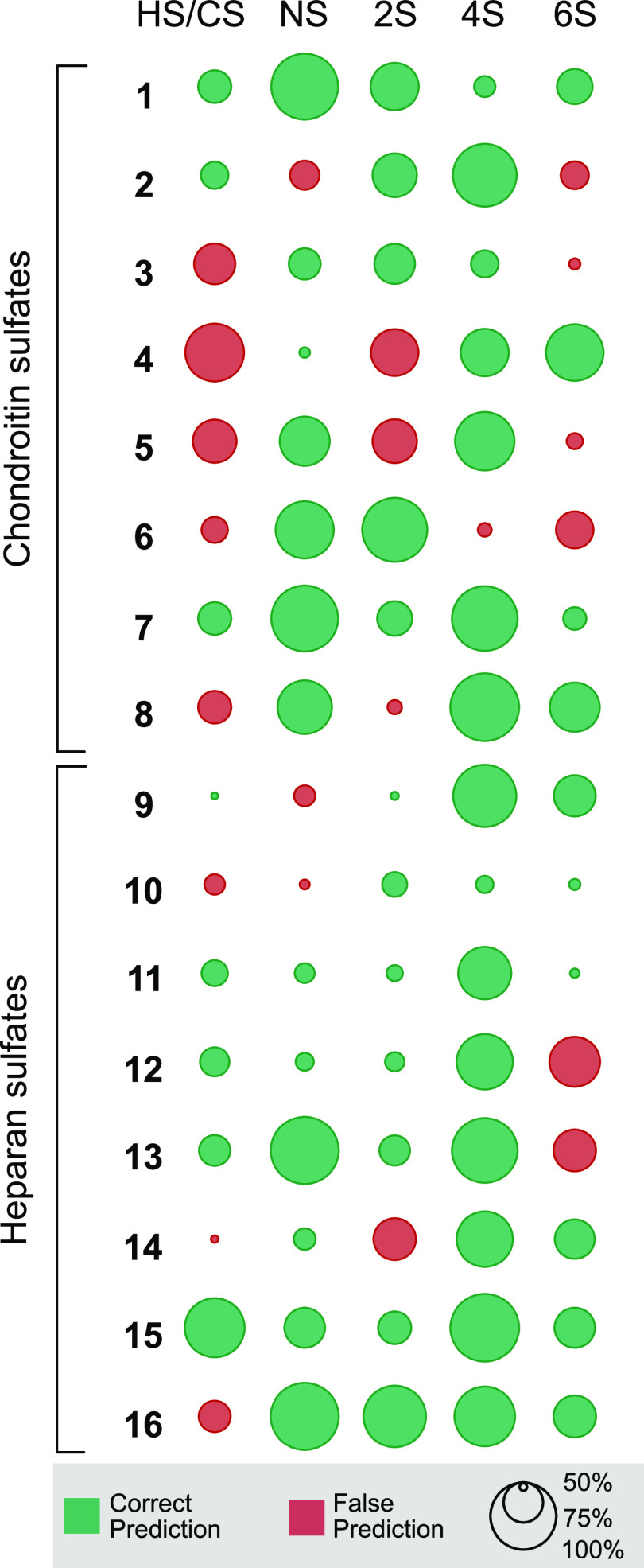
Prediction outcome and confidence for disaccharide classifications in the complete combinatorial space of excluding one sample from the base set. The prediction is for HS/CS, and the presence or absence of *N*-, 2-*O*-, 4-*O*-, and 6-*O*-sulfation (NS, 2S, 4S, and 6S, respectively).

In the joined data set of disaccharides and tetrasaccharides, the set of HS and CS disaccharides presented the purest, most complete, and most informative subset based on the number of unique sulfation patterns covered. However, as RF performance correlates with the size of the data set, training runs were initially focused on the performance of the algorithm for training sets smaller than 16 (the number of disaccharides). In this first step, training sets containing 15 disaccharides were assembled to evaluate the classification outcome in predicting the structure (HS or CS) and the presence or absence of *N*-, 2-*O*-, 4-*O*-, and 6-*O*-sulfation. This yielded a total of 80 models trained to cover the complete combinatorial space (Figure 4). To assess the impact of the training set on the model robustness, a prediction score was defined that averages the number of correct classifications across all excluded samples (see Supporting Information, Model Evaluation). The individual prediction scores were subsequently used to evaluate the overall prediction score over all training sets. The models predicted the correct structural motifs in 73% of the cases. High prediction accuracies were achieved in classification tasks for 4-*O*- and *N*-sulfation, with prediction scores of 94% and 81%, respectively. The feature with the best prediction scores (4-*O*-sulfation) classified all structures correctly, except for **6**, where the algorithm missed the spectral traits of the present 4-*O*-sulfation. Lower prediction accuracies were observed in predicting 2-*O*-sulfation (73%) and 6-*O*-sulfation (63%). In the case of 6-*O*-sulfation (with the worst prediction score among sulfation motifs), the structures **2**, **5**, and **6** were incorrectly assigned as 6-*O*-sulfated molecules, while the structures **3**, **12**, and **13** were falsely labeled as non-6-*O*-sulfated molecules. Similar accuracies had already been reported for functional group prediction from FTIR spectra of small organic molecules, with the accuracy depending on the functional group in question.^46^ Generally, high prediction accuracy is expected for *N*-sulfation due to the absence of the amide vibrations, thus rendering the 1200–1700 cm^–1^ range silent. In comparison, the lowest prediction accuracy is expected to be associated with HS/CS classifications, since HS and CS can potentially have varying monomer units and associated sulfation patterns, yielding nondiagnostic vibrational modes. This leads to more complex and ambiguous decision rules in the RF classifier. In the trained models, the classification for HS/CS remained inconclusive; the challenge of correctly classifying such structural differences is also reflected in the number of features selected to train the RF classifiers, with the highest feature count observed for HS/CS classifications.

The majority of features used in predicting *N*-sulfation were between 1200 and 1700 cm^–1^ (Table S8), covering the complete range of amide vibrations. The lack of amide vibrations in *N*-sulfated HS disaccharides resulted in strong decision rules in individual decision trees (i.e., intensities of ≈0 for *N*-acetylation). However, this spectral region is also populated by weak R–CH=CH–R and red-shifted carbonyl C=O stretching modes, which could give rise to false predictions. Based on Figure 4, an overall better prediction accuracy is observed for HS samples than for CS samples. This is due to the fact that in CS, sulfation at the 2-*O*, 4-*O*, and 6-*O* position must be predicted from vibrational modes in the range of 1150–1400 cm^–1^, whereas this region is only used to predict 2-*O*- and 6-*O*-sulfation in HS, allowing for better distinction. It is important to consider that the prediction confidence for false predictions was generally lower than for correct predictions, indicating that upon increase of the training set size, classification performance can likely be improved as further information in this region is added.

**Figure 5:**
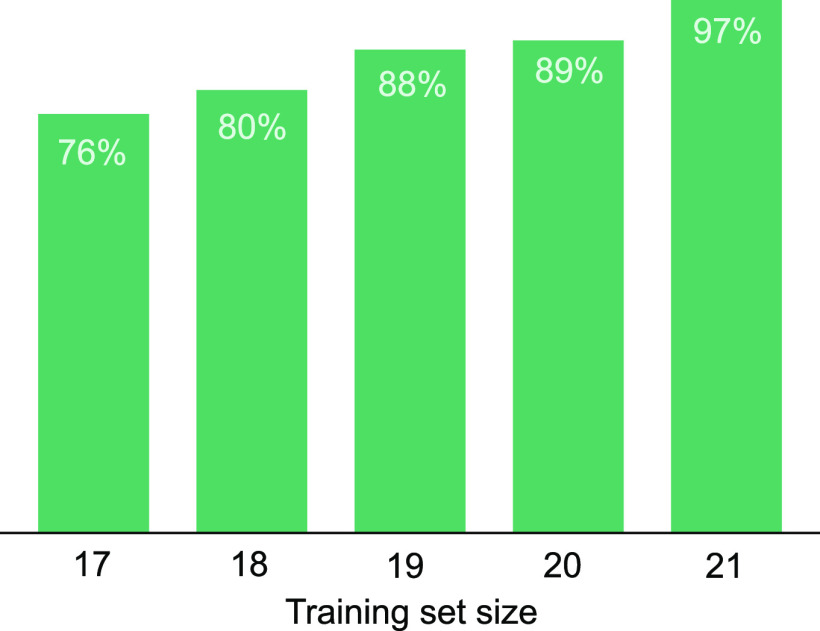
Prediction score as a function of the training set size. For each training set, RF classifiers were trained for the five classification tasks. The prediction outcome was averaged over all classifications and all training sets in the respective subset. The subsets were obtained by combinatorially adding tetrasaccharides to the disaccharide base set.

**Figure 6:**
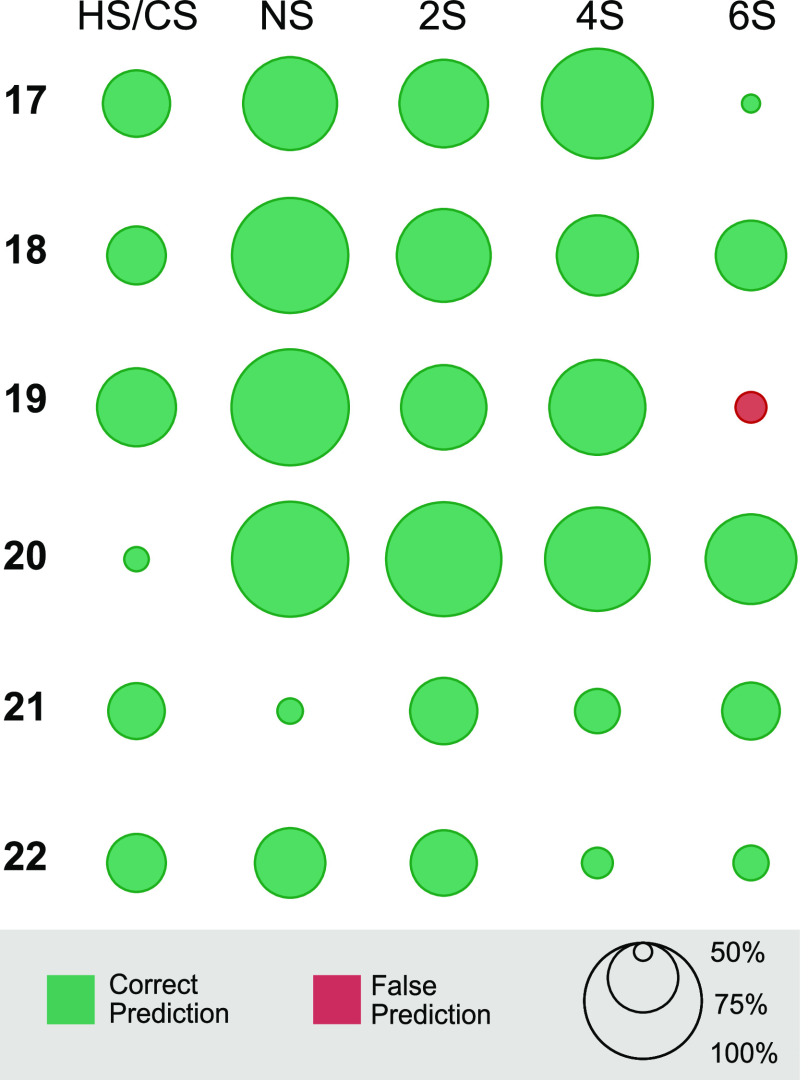
Prediction outcome and confidence in *X*_*m*=21_ training sets tested against the excluded sample. The classification outcomes are HS/CS, and the presence or absence of *N*-, 2-*O*-, 4-*O*-, and 6-*O*-sulfation (NS, 2S, 4S, and 6S, respectively).

To evaluate the influence of the training set size on the prediction score, IR spectra of tetrasaccharide samples **17**–**22** were systematically added to the training set. For model training, the full set of disaccharide IR spectra presented the base set of information and were always included. The tetrasaccharide samples were different not only in terms of the number of disaccharide building blocks, but also because of the presence of the aminopentyl linker. Therefore, a “label” feature was introduced to mark the difference between di- and tetrasaccharides by accounting for C–H and C–N deformations of the linker that were not yet part of the training set. By combinatorially adding tetrasaccharides to the base set, 62 unique training sets (*X*_*m*=17_ = 6, *X*_*m*=18_ = 15, *X*_*m*=19_ = 20, *X*_*m*=20_ = 15, *X*_*m*=21_ = 6) were assembled, leading to a total of 310 models trained for the five structural motifs. To evaluate the classification performance, the prediction score was calculated against the excluded tetrasaccharide samples and averaged over all predictions. Figure 5 shows that with increasing training set size, the prediction accuracy expectedly increased and approached a maximum prediction score of 97% for training sets with *m* = 21 samples. The RF classifiers were trained with bootstrap aggregation, i.e., only a subset of samples in the training set was used for growing the decision tree. This led to more diverse subsets due to the increased training set size, resulting in a minimized out-of-bag error rate in testing against the out-of-bag samples.^26,47^ A smaller out-of-bag error rate directly translates to higher prediction scores in evaluation runs against unknown samples,^24^ which is in agreement with the observations made in our classifications. In *X*_*m*=21_ training sets, HS, NS, 2S, and 4S structural motifs were predicted correctly across all excluded samples, with only a single classification for 6-*O*-sulfation failing with a confidence of 60% (Figure 6). The selected features for 6-*O*-sulfation were the “label” feature and wavenumber bins at 1122, 1212, 1467, 1587, 1632, and 1737 cm^–1^. Sample **19** is structurally related to samples **17**, **18**, and **20**, with the sulfation at the 6-*O* position being located on a GlcNAc unit. Comparing the IR spectra of samples **17** and **18** to the IR spectrum of sample **19**, clear intensity differences above 1450 cm^–1^ are visible, which was already linked to the presence of the terminal IdoA-GlcNAc6S-L1 building block for samples **19** and **20**.^14^ Therefore, by removing sample **19** from the data set, ambiguity was likely introduced into the model. In comparison, the model trained against sample **20** used the feature “charge” and wavenumber bins of 1212, 1287, and 1467 cm^–1^ for 6-*O*-sulfation, effectively avoiding the regions of intensity differences between the diastereomers, which yielded the overall correct prediction and higher prediction confidence (Table S9).

Although the “label” feature was not selected in all models (the evolutionary feature selection strategy employed here only yields local optima), it provided an unambiguous pathway to identify samples as HS. Since the “label” feature was not used in all RF classifiers, we believe that including this information may not be necessary. This is important from the standpoint of sample preparation, and for moving toward an analytical technique to predict structural motifs in truly unknown GAGs.

**Figure 7:**
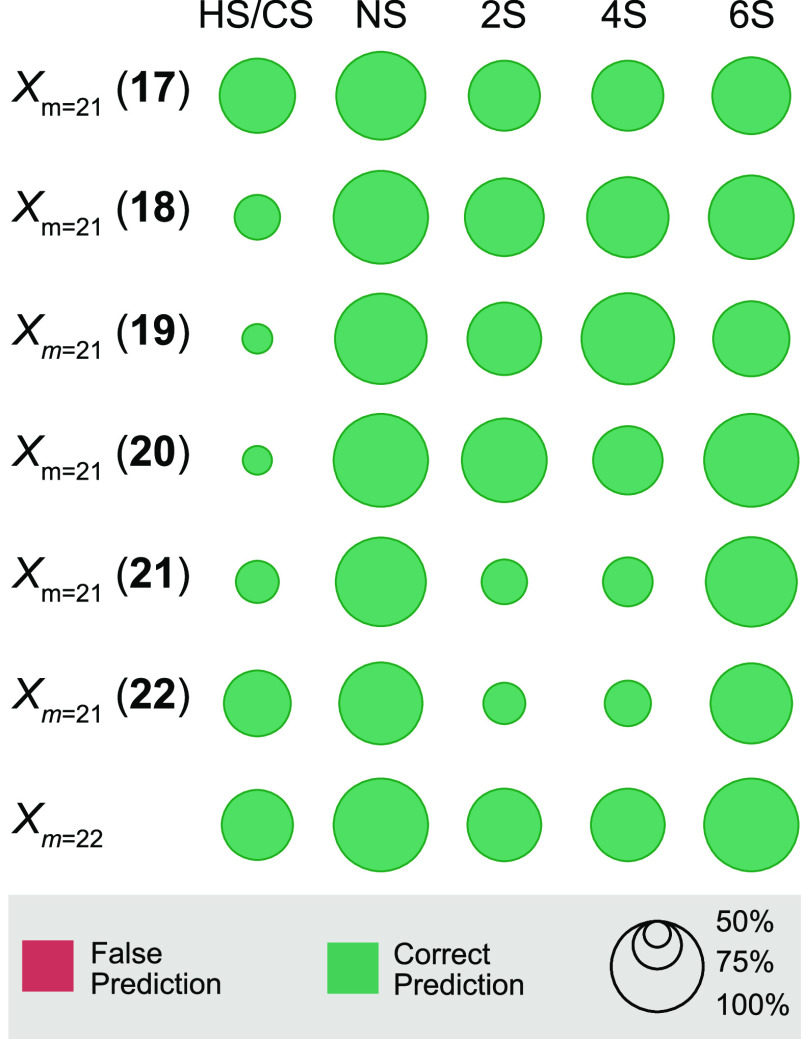
Prediction accuracy and probability of predicting the structural motifs of hexasaccharide 23 from *X*_*m*=21_ and *X*_*m*=22_ training sets. The sample in parentheses marks the tetrasaccharide that was not included in the training set. The prediction outcomes are HS/CS, and the presence or absence of *N*-, 2-*O*-, 4-*O*-, and 6-*O*-sulfation (NS, 2S, 4S, and 6S, respectively). Overall best prediction confidence was obtained for the *X*_*m*=22_ training set.

To assess the robustness of the method, we further extended the set of excluded samples by a hexasaccharide with the so far unknown *p*-methoxyphenyl linker. Upon validation of *X*_*m*=21_ and *X*_*m*=22_ training sets, all models classified the structural motifs of the hexasaccharide correctly (Figure 7). This indicates that training sets consisting of merely 21 high-resolution IR spectra are sufficient to make high-confidence predictions about structural motifs in unknown GAGs. Therefore, RF classification models trained on a representative sample set of different sulfation motifs can provide a computationally fast method for an initial estimate of GAG class and sulfation characteristics that provides complementary information to conventional mass spectrometry and ion mobility-based techniques. More importantly, the overall observed prediction accuracy suggests that a diverse library of smaller synthetic oligosaccharide fragments is sufficient to predict structural motifs in larger oligosaccharides, which are not yet accessible by chemical synthesis. Eventually, the outlined approach has the potential to be implemented in other cryogenic action spectroscopy approaches (e.g., tagging spectroscopy), where cryogenic temperatures increase spectral resolution and purge higher-energy conformers.

## Conclusion

In this work, helium nanodroplet spectra were recorded and used in Random Forest classifications to predict the class (HS/CS) and sulfate positions (2-*O*-, 4-*O*-, 6-*O*-, and *N*-sulfation) of GAG oligosaccharides. Even though certain structural motifs can be directly inferred from the vibrational signatures, it was expected that machine learning will lead to further improvement in feature identification and, with that, facilitate the automated identification of GAG functional groups. The implementation of an RF workflow revealed that, on surprisingly small training sets with as low as 21 samples, high confidence predictions of GAG structural motifs can be made. More importantly, the data indicate that a training set of small synthetic reference compounds such as di- and tetrasacchrides is sufficient to reliably predict structural motifs in larger structures up to hexasaccharides. This implies that structural annotations do not necessarily require an extensive set of synthetic standards to cover the full structural space. Instead, a few smaller, synthetically accessible molecules can be used to train the model for the structural prediction of larger, more complex GAG oligosaccharides, which are often not accessible by chemical synthesis. As a result, cryogenic gas-phase IR spectroscopy in combination with RF has exceptional potential to sequence larger GAG oligosaccharides to full-length GAG chains and serves as a blueprint for the analysis of other biomolecules, such as metabolites. In conjunction with classical ion-mobility and mass-spectrometry-based techniques, this has important implications for the understanding of GAG binding sequences, which can aid in the design and efficacy enhancement of novel and existing pharmaceuticals that target GAG structural motif patterns for disease treatment.

## Supporting Information Available

Additional sample identification, spectra, software descriptions, and model information (PDF)
