# Supplementary material for: Predicting Structural Motifs of Glycosaminoglycans using Cryogenic Infrared Spectroscopy and Random Forest
Source: J Am Chem Soc. 2023 Mar 31;145(14):7859–68. doi: 10.1021/jacs.2c12762 (PMC10103134; doi:10.1021/jacs.2c12762)
Supplement: Supplementary file 1 — ja2c12762_si_001.pdf [file ja2c12762_si_001.pdf]

Supporting Information

Predicting Structural Motifs of Glycosaminoglycans using Cryogenic Infrared Spectroscopy and Random Forest

Jerome Riedel[1,2], Maike Lettow[1,2], Márkó Grabarics[1,2], Michael Götze[1,2], Rebecca L. Miller[3], Geert-Jan Boons[4,5], Gerard Meijer[2], Gert von Helden[2], Gergo Peter Szekeres[1,2], Kevin Pagel[1,2]

[1]Freie Universität Berlin, Department of Biology, Chemistry, and Pharmacy, 14195 Berlin, Germany

[2]Fritz-Haber-Institut der Max-Planck-Gesellschaft, Department of Molecular Physics, 14195 Berlin, Germany

[3]University of Copenhagen, Copenhagen Center for Glycomics, Department of Cellular and Molecular Medicine, Copenhagen N 2200, Denmark

[4]Utrecht University, Bijvoet Center for Biomolecular Research, 3584 CG Utrecht, Netherlands

[5]University of Georgia, Complex Carbohydrate Research Center, Athens, GA, USA 30602-0002

Contents

1 Glycosaminoglycan labels **3**

2 Chondroitin sulfate spectra **4**

3 Heparan sulfate tetrasaccharide spectra **5**

4 Computational Details **6**

List of Tables

S1 Molecular structure labels 3

S4 Random Forest parameters 8

S5 Evolutionary feature selection parameters 8

S8 Feature selection for disaccharide predictions 10

S9 Feature selection for tetrasaccharide predictions 15

List of Figures

S2 The spectra of chondroitin sulfate disaccharides **1-8** in the range of 1000-1800 cm^{-1}. The spectra of the same species pre-processed with a different chain of algorithms are discussed in reference [1]. 4

S3 The spectra of heparan sulfate tetrasaccharides **17-20** in the range of 1000-1800 cm^{-1}. The spectra of the same species pre-processed with a different chain of algorithms are discussed in reference [2]. 5

S6 Prediction score as a function of the binning width 9

S7 Prediction score as a function of the number of estimators in the RF model 9

1 Glycosaminoglycan labels

Table S1: Labels of the different di-, tetra-, and hexasaccharides. Tetrasaccharides have an aminopentyl linker (L1). The hexasaccharide has a *p*-methoxyphenyl linker (L2).

Label	Structure	m/z
**1**	ΔUA**2S**β1-3GalNAc**4S6S**	205
**2**	ΔUA**2S**β1-3GalNAc**4S**	269
**3**	ΔUA**2S**β1-3GalNAc**6S**	269
**4**	ΔUAβ1-3GalNAc**4S6S**	269
**5**	ΔUA**2S**β1-3GalNAc	458
**6**	ΔUAβ1-3GalNAc**4S**	458
**7**	ΔUAβ1-3GalNAc**6S**	458
**8**	ΔUAβ1-3GalNAc	378
**9**	ΔUA**2S**β1-4Glc**NS6S**	191
**10**	ΔUA**2S**β1-4GlcNAc**6S**	269
**11**	ΔUA**2S**β1-4Glc**NS**	247
**12**	ΔUAβ1-4Glc**NS6S**	247
**13**	ΔUA**2S**β1-4GlcNAc	458
**14**	ΔUAβ1-4GlcNAc**6S**	458
**15**	ΔUAβ1-4Glc**NS**	416
**16**	ΔUAβ1-4GlcNAc	378
**17**	GlcAβ1-4GlcNAc**6S**α1-4GlcAβ1-4GlcNAc**6S**α1-4-L1	510
**18**	IdoAβ1-4GlcNAc**6S**α1-4GlcAβ1-4GlcNAc**6S**α1-4-L1	510
**19**	GlcAβ1-4GlcNAc**6S**α1-4IdoAβ1-4GlcNAc**6S**α1-4-L1	510
**20**	IdoAβ1-4GlcNAc**6S**α1-4IdoAβ1-4GlcNAc**6S**α1-4-L1	510
**21**	GlcAβ1-4Glc**NS6S**α1-4IdoAβ1-4Glc**NS6S**α1-4-L1	273
**22**	IdoAβ1-4Glc**NS6S**α1-4GlcAβ1-4Glc**NS6S**α1-4-L1	273
**23**	GlcNAc**6S**α1-4GlcAβ1-4GlcNAc**6S**α1-4GlcAβ1-4GlcNAc**6S**α1-4GlcAβ1-4-L2	499

2 Chondroitin sulfate spectra

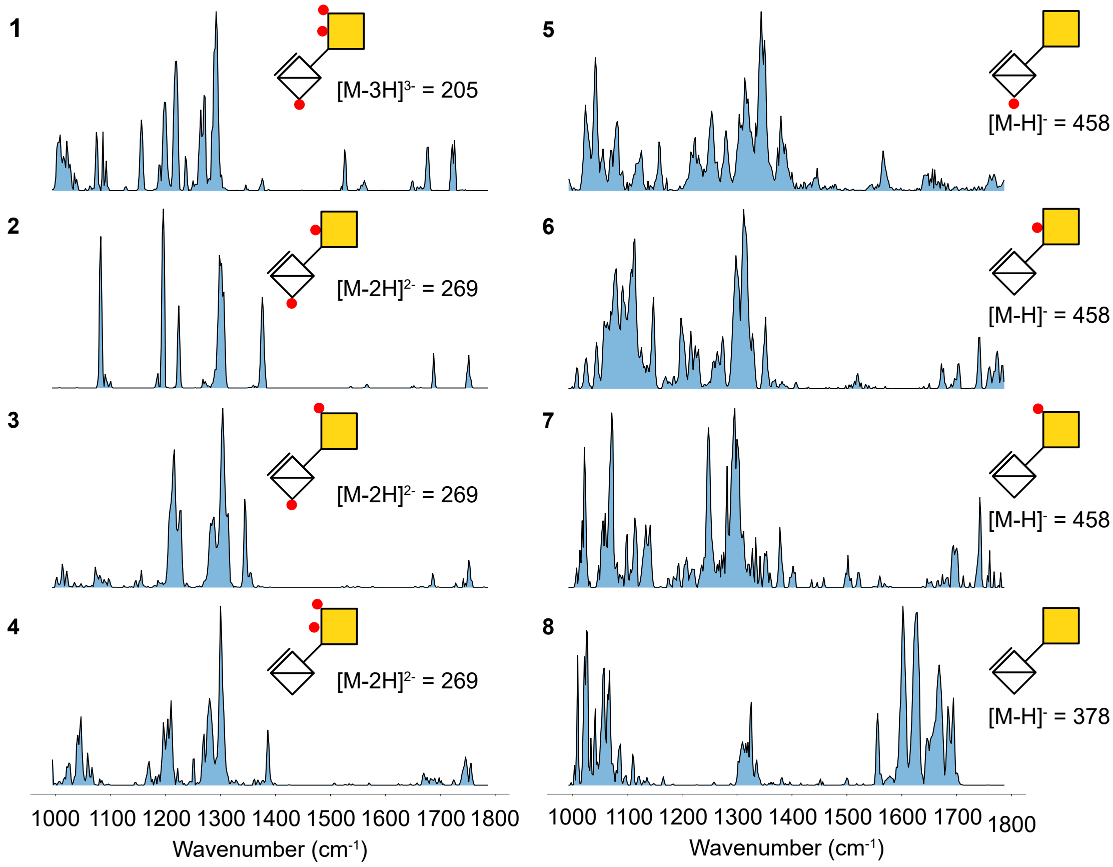

Figure S2: The spectra of chondroitin sulfate disaccharides **1**-**8** in the range of 1000-1800 cm^{-1}. The spectra of the same species pre-processed with a different chain of algorithms are discussed in reference [1].

3 Heparan sulfate tetrasaccharide spectra

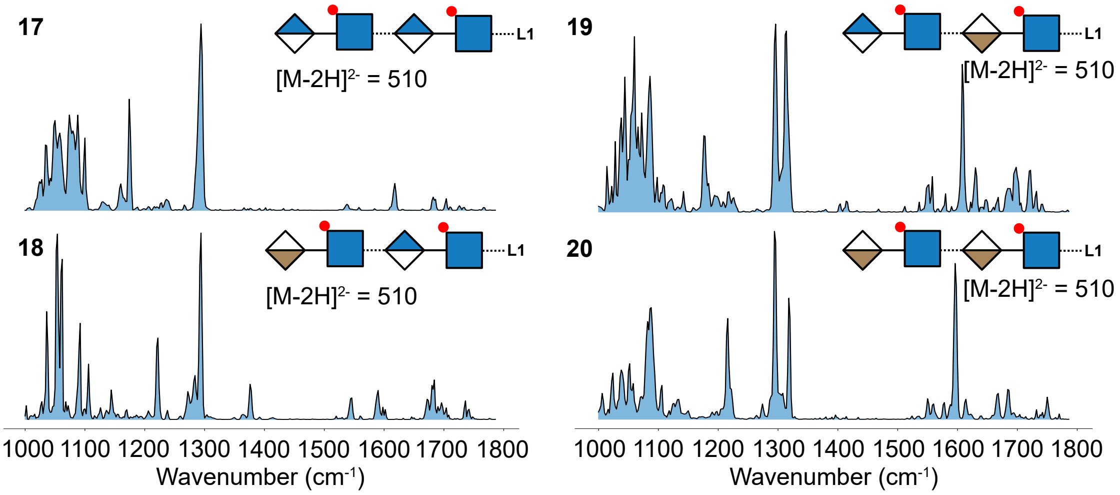

Figure S3: The spectra of heparan sulfate tetrasaccharides **17-20** in the range of 1000-1800 cm^{-1}. The spectra of the same species pre-processed with a different chain of algorithms are discussed in reference [2].

4 Computational Details

Data Pre-Processing

In an initial step before training, all spectra were scaled to account for differences in laser energy across the measurement regime, i.e., spectral regions measured at lower laser energies must be adjusted in comparison to those measured at higher laser energies. Therefore, IR intensities were normalized for the repetition rate and energy of the laser. After scaling, spectra were aligned on a commonly shared wavenumber axis by first order linear interpolation. In order to minimize the number of features in the spectrum before feature engineering, a histogram representation of the spectra was chosen. Given a histogram bin with left and right bin edges $\tilde{\nu}_l$ and $\tilde{\nu}_r$, the bin intensity is obtained by numerical integration of the IR trace between the bin edges. The size of the bin window was subjected to a hyperparameter search and determined to yield optimal results for bin window sizes of $15\,\mathrm{cm}^{-1}$ ($\pm 7.5\,\mathrm{cm}^{-1}$, see Figure S6). After spectral binning, bin intensities were normalized to the [0, 1] range.

Model Training

Classification of structural motifs was based on a Random Forest (RF) classifier trained on the pre-processed spectra. The computational implementation of the RF classifier was provided by *scikit-learn*, which uses the classification and regression tree (CART) algorithm for training.[3, 4] At every node level of growing the decision trees in the RF model, $\sqrt{k}$ features were considered, where k is the number of features in the training set after feature engineering. The splitting criterion was set to use the entropy formulation (information gain). It has been shown that the entropy formulation, albeit differences are small, produces more balanced trees than the Gini impurity, which might lead to improved feature selection.[3, 5] Cross-validation during model training was performed using a Leave-One-Out (LOO) approach. For feature selection, a genetic algorithm provided by *sklearn-genetic-opt* was utilized. The *GAFeatureSelectionCV* class provides a wrapper around the estimator, i.e., the RF classifier as outlined above.[6] Feature selection is performed using the $\mu+\lambda$ evolutionary optimization strategy.[6, 7] From a parent generation of μ individuals a new offspring generation with $\mu+\lambda$ individuals is generated. In addition to selecting individuals according to their fitness, a λ-tournament selection is performed, for which it is guaranteed that the λ-best individuals will be present in

the offspring generation. The population is evolved to a maximum of 40 generations or when convergence has been reached within five generations based on the population mean fitness. For a comprehensive summary of the algorithms applied here, the reader is referred to Eiben and Smith.[7] An overview of the different arguments defining the execution behaviour of the evolutionary algorithm in the feature engineering class is given in Table S5.

Model Evaluation

For model assessment, the performance (prediction score) of a binary classification model M_c for a structural motif c was evaluated against a set of augmented spectra $\mathbf{S}$ (i.e. addition of charge, sulfation count, etc.) that were excluded (holdout dataset) from the training library. Every spectrum $s \in \mathcal{S}$ is uniquely mapped to a glycosaminoglycan g with known structural motifs. We define a molecular evaluation function E for a molecule g with spectrum s as a function that, given a structural motif c, returns a boolean value whether c is present in g. A prediction score P for a structural motif c can then be calculated by averaging the prediction outcome over all excluded spectra

$$P_{\mathcal{S}}(c) = \frac{1}{|\mathcal{S}|} \sum_{\mathcal{S}} I(E(g,c) = M_c(s)), \tag{1}$$

where I is 1 if the equality holds, otherwise 0. The averaged prediction score $\bar{P}$ over the set of structural motifs $\mathcal{C}$ can then be calculated as follows

$$\bar{P}_{\mathcal{S}} = \frac{1}{|\mathcal{C}|} \sum_{\mathcal{C}} P_{\mathcal{S}}(c). \tag{2}$$

The prediction confidence T of the classification is determined as the ratio of decision trees t voting for spectrum s to belong to category c divided by the number of decision trees N_T in the model.

$$T(c) = \frac{1}{N_t} \sum_{i=1}^{N_t} I(t_i(s) = c), \tag{3}$$

where $t_i(s)$ evaluates the classification of the i-th decision tree in the RF model.

Table S4: Parameters provided in the constructor call of the *RandomForestClassifier* instance as provided by *scikit-learn.*[4] Listed are parameters used in the construction of trees. The number of estimators was derived from iterative testing (see Figure S7).

Parameter	Value
n_estimators	200
criterion	entropy
max_depth	None
min_samples_split	2
min_samples_leaf	1
min_weight_fraction_leaf	0
max_features	sqrt
max_leaf_nodes	None
min_impurity_decrease	0
bootstrap	True
max_samples	None
class_weight	None

Table S5: Selected parameters used in the constructur call of the *GAFeatureSelectionCV* instance.[6] *estimator* argument is a Random Forest classifier as obtained from *scikit-learn.*[4] Parameters of the classifier can be found in Table S4.

Parameter	Value
estimator	*RandomForestClassifier*
cv	None
population_size	150
generations	40
crossover_probability	0.8
mutation_probability	0.1
tournament_size	3
elitism	True
max_features	40
scoring	accuracy
criteria	max

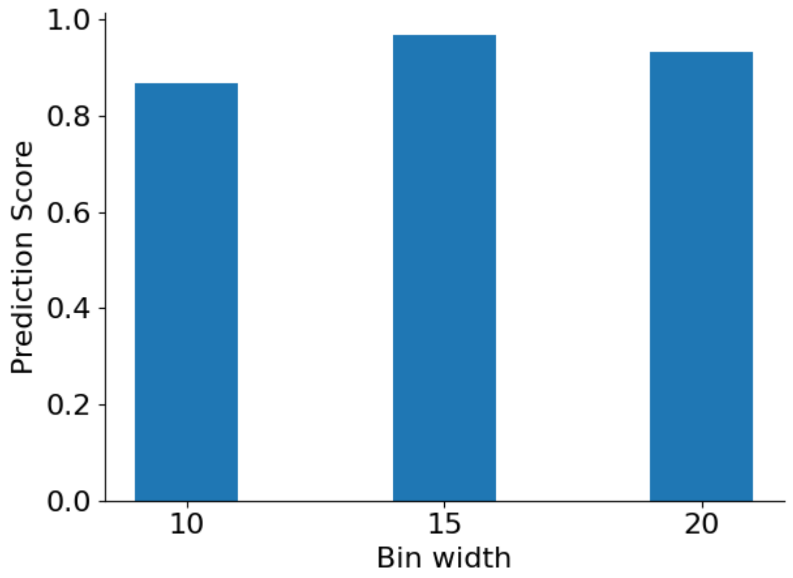

Figure S6: Prediction score as a function of the bin window width. Here, the average prediction score is shown for the different structural motifs for models trained on $X_{m=21}$ training sets.

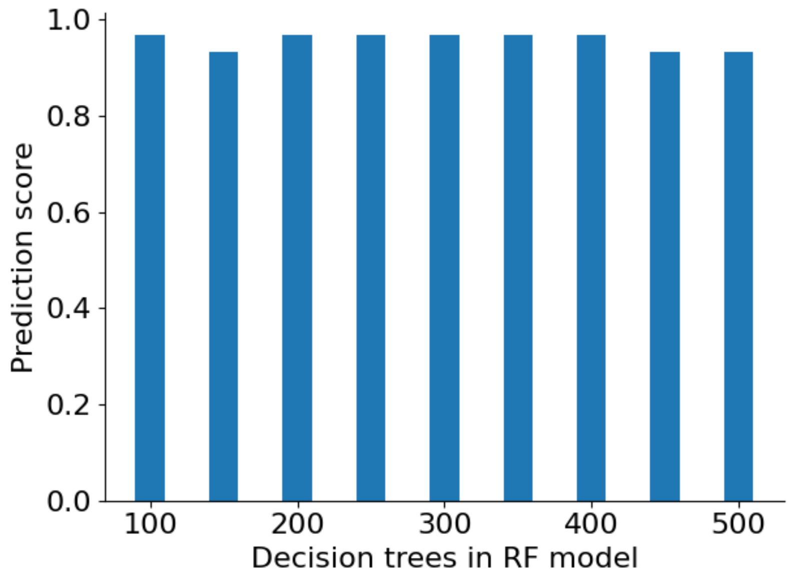

Figure S7: Prediction score as a function of the number of estimators in the RF model. Here, the average prediction score is shown for the different structural motifs for models trained on $X_{m=21}$ training sets.

Table S8: Selected features for the different models and their respective structural motif prediction (only disaccharides). "Charge" and "NSulfates" classified the charge state and number of sulfates present in the disaccharide, respectively.

Excluded sample	Structural motif	Selected features
**13**	HS	Charge, NSulfates, 1017, 1107, 1122, 1137, 1182, 1227, 1287, 1362, 1467, 1482, 1557, 1572, 1617, 1692, 1707, 1722
**16**	HS	1047, 1062, 1227, 1302, 1482, 1572, 1587, 1707, 1752, 1767
**3**	HS	1017, 1122, 1542, 1572
**5**	HS	NSulfates, 1017, 1077, 1212, 1347, 1362, 1407, 1482, 1692, 1782
**4**	HS	NSulfates, 1077, 1227, 1452, 1617
**6**	HS	1182, 1362, 1482, 1572, 1617
**2**	HS	1077, 1467, 1512, 1527, 1572, 1647
**14**	HS	NSulfates, 1062, 1227, 1362, 1572, 1707
**7**	HS	1137, 1227, 1287, 1572, 1602, 1692
**9**	HS	Charge, NSulfates, 1047, 1137, 1227, 1362, 1497, 1527, 1542, 1572, 1587
**1**	HS	1212, 1302, 1482, 1527, 1542, 1557, 1572

**10**	HS	1032, 1047, 1362, 1542, 1572, 1737
**15**	HS	NSulfates, 1227, 1542, 1572, 1692
**11**	HS	Charge, 1122, 1137, 1302, 1362, 1452, 1542, 1572, 1632, 1692
**12**	HS	1122, 1152, 1212, 1362, 1467, 1482, 1512, 1572, 1662, 1767
**8**	HS	1212, 1227, 1242, 1287, 1332, 1407, 1437, 1467, 1482, 1512, 1542, 1572, 1602, 1632, 1692, 1752
**6**	NS	1137, 1317, 1557, 1572, 1692
**7**	NS	1017, 1092, 1152, 1557, 1677, 1692
**8**	NS	1032, 1512, 1557, 1572, 1662
**1**	NS	1347, 1512, 1557, 1572
**16**	NS	1107, 1227, 1512, 1557, 1572, 1692
**12**	NS	Charge, 1152, 1512, 1542, 1572, 1737
**5**	NS	1212, 1227, 1347, 1467, 1527, 1542, 1782
**2**	NS	1467, 1482, 1527, 1677, 1692
**3**	NS	1437, 1512, 1542, 1572
**13**	NS	1512, 1557, 1692

**10**	NS	1212, 1227, 1392, 1422, 1557, 1572, 1692, 1707, 1722, 1782
**14**	NS	1017, 1047, 1212, 1242, 1287, 1542, 1632
**4**	NS	1047, 1182, 1497, 1542, 1557
**11**	NS	NSulfates, 1452, 1542, 1587, 1677, 1782
**9**	NS	1017, 1542, 1677
**15**	NS	1017, 1182, 1242, 1272, 1542, 1677, 1692
**10**	2S	1017, 1047, 1137, 1242, 1272, 1347, 1737
**13**	2S	1047, 1137, 1227, 1332, 1362, 1572, 1602
**4**	2S	1047, 1077, 1122, 1137, 1257, 1332, 1392, 1677
**9**	2S	1047, 1392, 1482, 1752
**5**	2S	1047, 1302, 1377, 1662
**14**	2S	1047, 1062, 1167, 1212, 1332, 1407, 1482, 1527, 1557, 1692, 1737, 1782
**15**	2S	1047, 1182, 1347, 1452, 1752, 1782
**2**	2S	1047, 1137, 1197, 1302, 1317, 1422, 1542, 1557, 1662, 1782

**3**	2S	1047, 1197, 1302, 1332, 1392, 1452, 1572, 1677, 1692, 1737, 1752, 1782
**8**	2S	1332, 1407
**12**	2S	1047, 1332, 1422, 1437, 1722
**7**	2S	1047, 1182, 1662
**16**	2S	1047, 1347, 1482, 1662
**1**	2S	1047, 1137, 1242, 1332, 1497, 1587, 1662, 1692
**11**	2S	1047, 1137, 1782
**6**	2S	1047, 1347
**16**	4S	1197, 1527, 1617, 1632
**3**	4S	1197, 1392, 1437, 1497, 1527, 1572, 1602, 1692
**1**	4S	1182, 1197, 1542
**6**	4S	1182, 1197, 1467
**14**	4S	1197, 1212, 1317, 1347, 1482, 1527
**8**	4S	1197
**10**	4S	Charge, 1167, 1197, 1242, 1317
**15**	4S	1197
**11**	4S	NSulfates, 1137, 1197, 1272, 1362, 1452
**2**	4S	1197
**7**	4S	1122, 1197, 1452, 1527
**12**	4S	1182, 1197, 1317, 1392, 1542

**13**	4S	1182, 1197, 1437, 1617
**4**	4S	1197, 1467, 1602
**9**	4S	Charge, 1107, 1197, 1227, 1437
**5**	4S	1197, 1287, 1302, 1332, 1347, 1437, 1497, 1572, 1587
**6**	6S	1212, 1287, 1332, 1422, 1512, 1632, 1737
**10**	6S	1182, 1212, 1287, 1422, 1482, 1587, 1617, 1692, 1737
**4**	6S	1212, 1227, 1287, 1467, 1737
**16**	6S	1212, 1467, 1737
**11**	6S	Charge, 1287, 1422, 1467, 1587, 1752
**15**	6S	1212, 1467
**7**	6S	NSulfates, 1122, 1212, 1287, 1467, 1587, 1737, 1782
**5**	6S	1017, 1197, 1212, 1287, 1407, 1527, 1572, 1737, 1782
**8**	6S	1182, 1212, 1227, 1467, 1737
**9**	6S	1407, 1467, 1482, 1572, 1602, 1632, 1722, 1737
**12**	6S	1032, 1047, 1287, 1467, 1662, 1722

**1**	6S	1152, 1212, 1272, 1422, 1467, 1527, 1737
**13**	6S	Charge, 1287, 1482, 1512, 1587
**3**	6S	1107, 1122, 1272, 1392, 1407, 1422, 1467, 1482, 1617, 1632, 1662, 1707, 1737
**14**	6S	1122, 1212, 1287, 1362, 1407, 1422, 1467, 1497, 1737
**2**	6S	1017, 1167, 1197, 1287, 1377, 1392, 1467, 1617, 1632, 1692, 1752

Table S9: Selected features for the different models and their respective structural motif prediction (tetrasaccharides added to the baseset of disaccharides). "Charge", "isLabeled", and "NSulfates" classified the charge state, label information, and number of sulfates of the di- and tetrasaccharides, respectively.

Add-on training samples	structural motif	selected Features
**18,21**	HS	isLabeled, 1077, 1362, 1422, 1542, 1617, 1647, 1692
**18**	HS	isLabeled, 1062, 1077, 1212, 1302, 1362, 1407, 1452, 1467, 1527, 1542, 1572, 1737, 1752, 1782
**17,18,19,22**	HS	1017, 1542, 1572, 1662
**17,20,21,22**	HS	1107, 1227, 1257, 1347, 1407, 1497, 1542

**18,19,21**	HS	isLabeled, 1302, 1467, 1527, 1542, 1587, 1602, 1617
**17,18,21**	HS	1167, 1287, 1362, 1407, 1467, 1482, 1602, 1677, 1752
**19,20,22**	HS	Charge, 1017, 1122, 1182, 1227, 1347, 1437, 1452, 1527, 1542, 1572, 1602, 1692
**21**	HS	NSulfates, 1152, 1227, 1302, 1542, 1572, 1602, 1692
**17,19**	HS	Charge, isLabeled, 1047, 1227, 1527, 1542, 1572, 1752
**20,22**	HS	NSulfates, isLabeled, 1062, 1167, 1197, 1227, 1242, 1287, 1302, 1332, 1362, 1467, 1512, 1527, 1542, 1572, 1587, 1602, 1707, 1752, 1782
**17,19,21,22**	HS	1032, 1047, 1152, 1212, 1227, 1287, 1362, 1467, 1617, 1692
**17,19,20,21**	HS	NSulfates, 1092, 1212, 1302, 1362, 1482, 1542
**22**	HS	1122, 1137, 1212, 1227, 1332, 1422, 1482, 1497, 1572, 1752

**17,18,20,22**	HS	1257, 1497, 1542, 1572, 1707
**17,20**	HS	NSulfates, isLabeled, 1122, 1542, 1572, 1752
**18,20,21,22**	HS	1017, 1257, 1407, 1482, 1542, 1602
**17,18,19,20**	HS	1197, 1242, 1542, 1572
**18,19,20**	HS	NSulfates, isLabeled, 1032, 1047, 1077, 1122, 1227, 1362, 1377, 1422, 1497, 1542, 1572, 1617
**17,21**	HS	1107, 1182, 1227, 1362, 1467, 1497, 1527, 1542, 1572, 1692
**18,19**	HS	Charge, 1122, 1467, 1602, 1752
**18,19,20,21**	HS	Charge, isLabeled, 1077, 1122, 1182, 1272, 1362, 1512, 1542, 1602, 1677, 1692, 1767
**17,19,22**	HS	isLabeled, 1152, 1182, 1227, 1542, 1572, 1602, 1632, 1707, 1752
**18,19,22**	HS	1017, 1092, 1107, 1137, 1332, 1512, 1542, 1572, 1752
**17,19,20,22**	HS	1122, 1182, 1272, 1467, 1542, 1572, 1587, 1602, 1617, 1692
**17,22**	HS	1047, 1182, 1332, 1542, 1572, 1617

**18,20,22**	HS	1122, 1182, 1347, 1452, 1467, 1482, 1497, 1527, 1542, 1617, 1662
**20**	HS	1047, 1122, 1167, 1197, 1212, 1242, 1377, 1527, 1572
**17,20,21**	HS	NSulfates, 1077, 1107, 1122, 1227, 1332, 1362, 1437, 1467, 1542, 1662, 1677, 1692
**17,18,19,20,22**	HS	1242, 1467, 1482, 1512, 1542, 1557, 1752
**19,21,22**	HS	isLabeled, 1437, 1527, 1572, 1617, 1707, 1752
**18,20**	HS	1122, 1467, 1602, 1752, 1767, 1782
**19**	HS	1047, 1122, 1137, 1182, 1212, 1227, 1257, 1302, 1407, 1437, 1467, 1542, 1557, 1572, 1692, 1722, 1752
**18,22**	HS	1077, 1362, 1467, 1542, 1692
**18,19,20,22**	HS	Charge, NSulfates, isLabeled, 1182, 1527, 1542, 1572, 1602

**17,18,21,22**	HS	NSulfates, isLabeled, 1032, 1107, 1137, 1167, 1197, 1272, 1317, 1347, 1362, 1407, 1467, 1542, 1572, 1587, 1602, 1617, 1662, 1677, 1692, 1752, 1767
**19,20,21,22**	HS	isLabeled, 1527, 1542, 1572
**19,22**	HS	NSulfates, 1017, 1122, 1182, 1197, 1212, 1227, 1302, 1422, 1437, 1452, 1527, 1542, 1572, 1602, 1707
**20,21**	HS	1167, 1227, 1362, 1437, 1542, 1587, 1602, 1692
**19,20,21**	HS	Charge, isLabeled, 1017, 1122, 1227, 1557, 1617
**18,19,21,22**	HS	1077, 1122, 1272, 1482, 1602, 1722, 1752, 1767
**18,19,20,21,22**	HS	NSulfates, isLabeled, 1017, 1242, 1317, 1362, 1437, 1527, 1572, 1602, 1617, 1677, 1692
**17,19,21**	HS	isLabeled, 1122, 1197, 1227, 1332, 1617, 1632, 1707, 1722

**17,20,22**	HS	1152, 1212, 1272, 1287, 1362, 1482, 1527, 1542, 1557, 1602, 1617, 1662, 1692
**17,18**	HS	Charge, 1212, 1242, 1407, 1467, 1542, 1572, 1587, 1707, 1752, 1767
**17,21,22**	HS	1167, 1182, 1212, 1227, 1467, 1542, 1572, 1692, 1737, 1752, 1767
**17,19,20,21,22**	HS	1062, 1107, 1182, 1302, 1347, 1362, 1392, 1407, 1467, 1512, 1527, 1542, 1572, 1602, 1617, 1632, 1692, 1737, 1752
**17,18,20,21**	HS	NSulfates, 1122, 1212, 1362, 1497, 1542, 1602, 1752
**21,22**	HS	isLabeled, 1122, 1182, 1212, 1227, 1272, 1302, 1347, 1362, 1527, 1572, 1602, 1752
**17,18,20**	HS	1137, 1182, 1242, 1257, 1272, 1362, 1422, 1482, 1542, 1572, 1752, 1767

**18,20,21**	HS	1062, 1122, 1137, 1212, 1437, 1467, 1542, 1602, 1617, 1692, 1752
**18,21,22**	HS	Charge, 1077, 1107, 1122, 1137, 1257, 1287, 1332, 1362, 1407, 1452, 1467, 1512, 1527, 1542, 1617, 1662, 1692, 1707, 1722
**17,18,19**	HS	1047, 1212, 1542, 1572, 1752, 1767
**20,21,22**	HS	isLabeled, 1137, 1227, 1242, 1422, 1527, 1542, 1572, 1602, 1632, 1692
**17,18,19,21,22**	HS	isLabeled, 1107, 1122, 1212, 1257, 1467, 1542, 1572, 1752, 1767
**17**	HS	1137, 1182, 1227, 1347, 1377, 1467, 1542, 1572, 1662, 1752, 1767, 1782
**17,18,19,21**	HS	1032, 1047, 1212, 1392, 1467, 1542, 1572, 1752
**17,19,20**	HS	1122, 1212, 1227, 1287, 1347, 1527, 1542, 1557, 1572, 1602
**19,20**	HS	1527, 1542, 1572

**17,18,20,21,22**	HS	1062, 1527, 1542, 1602, 1662, 1752
**19,21**	HS	isLabeled, 1077, 1122, 1182, 1227, 1287, 1452, 1467, 1662, 1707
**17,18,22**	HS	NSulfates, 1137, 1242, 1287, 1302, 1362, 1407, 1467, 1542, 1572, 1752, 1767
**17,18,19,20,21**	HS	NSulfates, 1032, 1047, 1122, 1152, 1212, 1617, 1722
**17,21,22**	NS	1152, 1242, 1542, 1692
**18,20,21**	NS	1197, 1542, 1557, 1692
**18,19,20,21,22**	NS	1092, 1542, 1557, 1662, 1707, 1737
**18,22**	NS	1287, 1377, 1557, 1692, 1737
**17,18,20,21**	NS	1392, 1527, 1542, 1557, 1662
**19,21**	NS	isLabeled, 1272, 1392, 1497, 1527, 1542, 1587, 1692
**17,19,20,22**	NS	1032, 1047, 1107, 1497, 1527, 1557, 1692
**17,18,20,21,22**	NS	1017, 1467, 1527, 1557, 1662, 1692
**18,21**	NS	1047, 1077, 1452, 1482, 1542, 1557, 1692
**20**	NS	1017, 1542, 1572
**18**	NS	1077, 1527, 1557, 1692

**20,21,22**	NS	Charge, 1137, 1197, 1227, 1332, 1542, 1572, 1692
**21**	NS	1227, 1512, 1542
**17,18,19,20,21**	NS	NSulfates, 1317, 1542, 1617, 1632, 1677
**17,20,22**	NS	1527, 1557, 1587, 1692
**17,18,22**	NS	isLabeled, 1032, 1302, 1602, 1677, 1692
**17,19,22**	NS	1017, 1062, 1527, 1572, 1692
**17,19,21,22**	NS	1227, 1287, 1332, 1347, 1482, 1527, 1557, 1692
**17,19**	NS	1137, 1242, 1272, 1392, 1482, 1542, 1557, 1692
**19,22**	NS	1122, 1272, 1362, 1512, 1527, 1557, 1572, 1707
**18,20,22**	NS	1122, 1272, 1377, 1422, 1497, 1512, 1572, 1692
**18,19,22**	NS	1137, 1227, 1527, 1557, 1572, 1602, 1722
**18,20**	NS	isLabeled, 1167, 1197, 1527, 1542, 1647
**17,18,19**	NS	1137, 1437, 1482, 1542, 1572, 1632, 1752
**17,20**	NS	isLabeled, 1047, 1227, 1242, 1302, 1362, 1527, 1542, 1602

**17,21**	NS	1197, 1542, 1557, 1692
**18,19**	NS	1032, 1527, 1557, 1692
**18,19,21**	NS	1242, 1347, 1377, 1422, 1527, 1557, 1692
**20,22**	NS	1017, 1212, 1527, 1542, 1692
**18,20,21,22**	NS	isLabeled, 1032, 1227, 1362, 1512, 1632, 1692
**17,18,19,21,22**	NS	1062, 1542, 1557, 1692, 1782
**20,21**	NS	Charge, 1077, 1092, 1137, 1257, 1512, 1542, 1557, 1692
**17,18**	NS	1557, 1572, 1677
**18,19,20**	NS	NSulfates, 1032, 1467, 1512, 1557, 1572, 1617, 1632
**18,21,22**	NS	1527, 1557, 1692
**17,18,19,21**	NS	1527, 1542, 1557, 1572, 1662, 1782
**17,18,21,22**	NS	1542, 1632, 1662, 1692
**17**	NS	isLabeled, 1302, 1422, 1482, 1542, 1557, 1572
**17,19,21**	NS	Charge, 1542, 1557, 1782
**17,20,21,22**	NS	1047, 1542, 1557, 1692
**17,18,20,22**	NS	1197, 1317, 1542, 1557, 1572
**19**	NS	1152, 1182, 1362, 1542, 1692, 1737
**17,22**	NS	1092, 1557, 1572, 1677

**18,19,20,22**	NS	1017, 1107, 1197, 1377, 1512, 1542, 1557, 1692
**17,19,20**	NS	1137, 1332, 1377, 1527, 1542
**19,20,21**	NS	Charge, 1377, 1527, 1542, 1557, 1632, 1692
**22**	NS	1482, 1527, 1557, 1692, 1722, 1752
**18,19,20,21**	NS	NSulfates, isLabeled, 1152, 1482, 1557, 1677, 1692
**17,18,19,22**	NS	1422, 1497, 1527, 1557, 1692
**17,18,19,20,22**	NS	1497, 1527, 1572, 1692, 1722
**17,18,20**	NS	1017, 1107, 1212, 1317, 1437, 1527, 1557, 1692
**21,22**	NS	1287, 1557, 1617, 1677, 1692, 1737
**18,19,21,22**	NS	isLabeled, 1167, 1302, 1392, 1677, 1692
**17,18,19,20**	NS	isLabeled, 1047, 1182, 1542, 1557, 1572
**19,20**	NS	1107, 1137, 1512, 1572, 1617, 1692, 1707
**17,18,21**	NS	1137, 1272, 1527, 1557, 1692
**17,20,21**	NS	1017, 1077, 1107, 1347, 1497, 1557, 1662, 1692

**19,21,22**	NS	Charge, isLabeled, 1332, 1347, 1557, 1617, 1677, 1692, 1752
**17,19,20,21,22**	NS	1032, 1092, 1557, 1677, 1692
**17,19,20,21**	NS	1227, 1542, 1662, 1677
**19,20,21,22**	NS	Charge, 1242, 1287, 1512, 1542, 1692
**19,20,22**	NS	1332, 1527, 1542, 1557, 1692
**18,21**	2S	1047, 1242, 1302, 1347, 1482, 1707
**17**	2S	1047, 1182, 1347
**17,18,22**	2S	1047, 1137, 1167, 1347, 1572
**18,19,21,22**	2S	1047, 1287, 1347, 1362, 1452, 1467, 1497, 1692
**17,19,20,22**	2S	Charge, 1047, 1242, 1467, 1482, 1572
**21,22**	2S	1047, 1257, 1332, 1452, 1782
**19,22**	2S	Charge, 1047, 1227, 1287, 1302, 1317
**17,20,21,22**	2S	1047, 1137, 1572, 1602, 1647, 1722, 1767
**17,18,20,22**	2S	1047, 1347
**17,18,19,21,22**	2S	1047, 1287, 1332, 1347
**18,19,22**	2S	1047, 1332, 1407, 1572, 1632

**17,18,19,21**	2S	1017, 1047, 1167, 1332, 1392, 1557, 1572, 1737
**17,19**	2S	1047, 1347
**17,18,19,22**	2S	1047, 1137, 1167, 1257, 1347
**17,18,19,20,21**	2S	Charge, 1017, 1047, 1287, 1377, 1512
**18,19,20,22**	2S	1017, 1047, 1347, 1377, 1617
**19,20**	2S	1047, 1332, 1482, 1512, 1542
**19,20,21,22**	2S	1047, 1287, 1377, 1632
**19**	2S	1017, 1047, 1347, 1617, 1782
**17,20,21**	2S	1047, 1347
**18,22**	2S	1047, 1377
**17,18,21**	2S	isLabeled, 1047, 1182, 1272, 1347
**17,18,19,20,22**	2S	Charge, 1047, 1317
**20,22**	2S	1047, 1242, 1347, 1362
**18,19,20**	2S	1047, 1182, 1302, 1392, 1662, 1677, 1692
**17,21,22**	2S	NSulfates, 1047, 1182, 1542
**19,20,22**	2S	1047, 1347
**17,19,20,21,22**	2S	1047, 1062, 1422, 1482, 1722, 1737
**18,20,21**	2S	NSulfates, 1047, 1317, 1347, 1752
**17,18**	2S	1047, 1212, 1332, 1347, 1437, 1587, 1647, 1662, 1782

**18,20**	2S	1047, 1182, 1467, 1557
**17,18,19**	2S	Charge, 1032, 1047, 1137, 1167, 1257, 1347
**17,18,20,21**	2S	1017, 1047, 1332
**18,19,20,21,22**	2S	1047, 1197, 1347, 1512, 1752
**17,19,20,21**	2S	1047, 1227, 1302, 1317, 1347
**17,19,22**	2S	isLabeled, 1047, 1317, 1437, 1482, 1587, 1692
**19,21**	2S	1047, 1182, 1227, 1242, 1347, 1467, 1572
**17,18,21,22**	2S	1047, 1212, 1332, 1437, 1707
**18,20,21,22**	2S	1047, 1662
**17,18,19,20**	2S	1047, 1437
**18,19,20,21**	2S	1047, 1257, 1302, 1317, 1347
**18,21,22**	2S	1182, 1347, 1392
**17,20,22**	2S	1047, 1332
**21**	2S	1017, 1047, 1332, 1392, 1587
**17,22**	2S	1047, 1182, 1242, 1332, 1392, 1482, 1752
**20**	2S	1047, 1077, 1197, 1227, 1242, 1347, 1482, 1767, 1782
**17,19,21,22**	2S	1047, 1182, 1392, 1437, 1482
**17,20**	2S	1047, 1332, 1782
**17,18,20,21,22**	2S	Charge, isLabeled, 1047, 1662, 1782

**17,18,20**	2S	NSulfates, 1047, 1077, 1137, 1167, 1212, 1257, 1272, 1347, 1542, 1677, 1737
**20,21**	2S	1047, 1182, 1392, 1452, 1557
**18,19,21**	2S	1047, 1182, 1407, 1677, 1737, 1767
**17,19,20**	2S	1047, 1077, 1092, 1167, 1317, 1332, 1347, 1377
**19,20,21**	2S	1047, 1332, 1347, 1437, 1677
**20,21,22**	2S	1047, 1197, 1347, 1482
**18,20,22**	2S	NSulfates, 1047, 1167, 1302, 1347, 1437
**18,19**	2S	Charge, 1047, 1602
**17,19,21**	2S	1047, 1452, 1467, 1482, 1497, 1572, 1767
**18**	2S	1047, 1482
**17,21**	2S	1047, 1182, 1347, 1482
**22**	2S	1047, 1182, 1392, 1452, 1482, 1632, 1677, 1752
**19,21,22**	2S	1047, 1272, 1287, 1332, 1482, 1587, 1632
**18,19,22**	4S	1182, 1197, 1377, 1422, 1437, 1482, 1542, 1572, 1587, 1707
**21,22**	4S	1197, 1482, 1602
**18,22**	4S	1197, 1542, 1572, 1602

**17,19,20,21**	4S	Charge, 1137, 1182, 1197, 1482, 1602
**18,19,21**	4S	1047, 1137, 1197, 1317, 1332, 1362, 1377, 1482, 1497, 1632, 1647, 1662
**18**	4S	1197
**18,19**	4S	1197
**17,19,21,22**	4S	1197, 1347, 1602
**17,20**	4S	1197, 1422, 1437
**20,21,22**	4S	1017, 1107, 1122, 1137, 1197, 1437, 1452, 1572
**19**	4S	1197
**17,18,20,21,22**	4S	1137, 1227, 1317, 1407, 1482, 1572
**19,20**	4S	1197, 1242, 1437, 1602
**17,18,19,20,21**	4S	1197, 1362, 1482, 1542, 1602
**18,19,20**	4S	1107, 1197, 1437, 1452, 1572, 1602
**17,19**	4S	1152, 1197, 1287, 1782
**20**	4S	1197, 1227, 1347, 1572, 1677
**17,18,22**	4S	1197, 1542, 1572, 1602
**17,18,19,20**	4S	1137, 1197, 1422, 1497, 1542, 1782
**17,18,19,22**	4S	1197, 1542, 1572, 1602, 1767
**17**	4S	NSulfates, 1197, 1572, 1722
**18,19,21,22**	4S	1197, 1437, 1572, 1602

**17,19,20,21,22**	4S	isLabeled, 1197, 1227, 1572, 1602, 1617
**19,20,21**	4S	1137, 1197, 1467, 1572, 1602
**17,18,20**	4S	1197, 1212, 1602
**18,20**	4S	1197, 1452
**17,19,22**	4S	1197, 1287, 1347, 1407, 1437, 1572, 1632
**17,20,21**	4S	1137, 1437, 1572, 1602
**18,20,21**	4S	NSulfates, isLabeled, 1197, 1347, 1602, 1707
**18,20,22**	4S	NSulfates, 1197, 1437, 1542, 1572
**17,21,22**	4S	Charge, 1197, 1467, 1542
**18,21,22**	4S	1197, 1602
**17,18,19,21**	4S	1377, 1437, 1572, 1602, 1782
**19,20,22**	4S	1107, 1137, 1197, 1572, 1587, 1647, 1782
**17,22**	4S	1032, 1197, 1602, 1662, 1782
**17,18,21,22**	4S	isLabeled, 1197, 1347, 1422, 1467
**17,19,21**	4S	Charge, 1047, 1107, 1122, 1152, 1197, 1227, 1377
**17,18,19,20,22**	4S	isLabeled, 1137, 1182, 1197, 1227, 1407, 1482, 1617, 1647, 1767
**17,18**	4S	1137, 1197, 1227, 1347, 1467, 1572

**20,21**	4S	1137, 1197, 1227, 1362, 1407, 1647, 1782
**17,20,21,22**	4S	1197, 1437, 1572, 1647, 1782
**18,21**	4S	NSulfates, 1047, 1197, 1287, 1347, 1437, 1602
**17,18,20,22**	4S	1197, 1302, 1407, 1482, 1572
**17,21**	4S	1137, 1197, 1407, 1437, 1527, 1572
**17,18,21**	4S	Charge, 1047, 1197, 1272, 1377, 1437, 1572, 1602
**17,18,20,21**	4S	1137, 1197, 1437, 1572, 1602
**19,21,22**	4S	1197, 1227, 1482, 1572, 1647
**17,18,19**	4S	1197, 1572
**17,18,19,21,22**	4S	1137, 1197, 1467, 1497, 1602, 1782
**21**	4S	isLabeled, 1167, 1197, 1272, 1362, 1437, 1542, 1602
**19,22**	4S	1152, 1197, 1437, 1542, 1572
**17,20,22**	4S	1197, 1212, 1332, 1347, 1497, 1572, 1602
**19,21**	4S	Charge, isLabeled, 1197, 1347, 1362, 1437, 1542, 1572, 1617, 1692, 1707

**20,22**	4S	1197, 1287, 1347, 1467, 1572
**17,19,20**	4S	1197, 1347, 1437
**18,19,20,21,22**	4S	Charge, isLabeled, 1197, 1317, 1452, 1572, 1587, 1767
**18,19,20,22**	4S	1017, 1077, 1122, 1197, 1302, 1347, 1377, 1572, 1647, 1782
**17,19,20,22**	4S	1197, 1332, 1437, 1572, 1602, 1662
**19,20,21,22**	4S	1197, 1602, 1782
**18,19,20,21**	4S	1137, 1197, 1317, 1362, 1617
**22**	4S	Charge, 1167, 1197, 1347, 1377, 1572
**18,20,21,22**	4S	1197, 1572, 1602
**17,18,20**	6S	1032, 1212, 1287, 1377, 1407, 1467, 1482, 1707, 1737
**17,20,21**	6S	1212, 1287, 1467, 1587, 1632, 1737
**18,21,22**	6S	1122, 1182, 1212, 1347, 1377, 1407, 1467, 1602, 1722, 1737
**17,19,21,22**	6S	1197, 1212, 1227, 1332, 1362, 1467, 1482, 1512, 1527, 1737
**17,18,19,21,22**	6S	Charge, 1212, 1287, 1467
**19,21**	6S	1182, 1197, 1212, 1287, 1527, 1617, 1677, 1782

**17,21,22**	6S	isLabeled, 1017, 1032, 1077, 1092, 1152, 1182, 1197, 1212, 1332, 1407, 1437, 1467, 1482, 1527, 1632, 1662, 1737, 1782
**17,19,22**	6S	1077, 1212, 1227, 1332, 1482, 1527
**17,18,19,20**	6S	1107, 1182, 1212, 1287, 1317, 1332, 1437, 1482, 1497, 1617, 1692, 1707, 1737, 1782
**20,21,22**	6S	1212, 1287, 1467, 1647, 1737
**18**	6S	1032, 1167, 1182, 1212, 1467, 1482, 1587, 1617, 1677, 1737
**20,22**	6S	1152, 1167, 1212, 1272, 1287, 1482
**18,20,21**	6S	1212, 1287, 1467
**19,20,21**	6S	1092, 1137, 1197, 1212, 1287, 1302, 1392, 1467, 1482, 1647, 1707, 1737
**17,19,20,21**	6S	isLabeled, 1092, 1197, 1212, 1227, 1347, 1362, 1377, 1407, 1467, 1737, 1782
**21**	6S	1152, 1212, 1287, 1392, 1407, 1467, 1587, 1737

**17,18,20,22**	6S	1062, 1122, 1212, 1287, 1467, 1482, 1617, 1737
**18,19,20**	6S	NSulfates, 1017, 1032, 1212, 1227, 1527, 1572, 1587, 1737
**17,19,21**	6S	Charge, 1287, 1392, 1467, 1497, 1737
**19**	6S	Charge, isLabeled, 1197, 1287, 1452, 1467, 1737
**17,19,20,21,22**	6S	isLabeled, 1212, 1227, 1467, 1542, 1647, 1737
**19,22**	6S	1017, 1182, 1197, 1212, 1467, 1497, 1677, 1737, 1752, 1767
**17,20**	6S	Charge, NSulfates, 1212, 1227, 1392, 1437, 1467, 1482, 1527, 1587, 1677, 1737, 1782
**22**	6S	1212, 1287, 1467, 1587, 1662
**17,18,21**	6S	isLabeled, 1092, 1212, 1467, 1632, 1737
**17,18,22**	6S	isLabeled, 1182, 1212, 1392, 1467, 1737, 1782
**18,21**	6S	1212, 1287, 1467, 1482, 1497, 1662
**18,20**	6S	Charge, 1092, 1212, 1287, 1482, 1497, 1587, 1632, 1737

**18,19,20,21**	6S	NSulfates, 1092, 1182, 1212, 1302, 1407, 1467, 1482, 1587, 1632, 1647, 1677, 1737
**18,19,20,22**	6S	isLabeled, 1212, 1272, 1287, 1407, 1482
**17,19,20**	6S	isLabeled, 1212, 1287, 1482, 1587, 1737, 1752
**17,18,20,21**	6S	1182, 1212, 1467, 1677, 1737
**17,20,21,22**	6S	1017, 1152, 1212, 1287, 1377, 1392, 1437, 1452, 1467, 1632, 1722, 1737
**18,19,20,21,22**	6S	1197, 1212, 1227, 1332, 1392, 1467, 1512, 1587, 1632, 1737
**20,21**	6S	1167, 1212, 1302, 1422, 1467, 1527, 1572, 1662, 1737, 1767
**17,18,20,21,22**	6S	isLabeled, 1122, 1212, 1467, 1587, 1632, 1737
**17,18,19,20,21**	6S	isLabeled, 1182, 1212, 1362, 1467, 1737, 1782
**17,18,21,22**	6S	NSulfates, 1212, 1467, 1587, 1617, 1677, 1722, 1737, 1752
**17,20,22**	6S	NSulfates, 1212, 1302, 1332, 1392, 1422, 1467, 1482, 1512, 1587, 1632, 1722, 1737

**21,22**	6S	1047, 1212, 1227, 1362,<br>1452, 1467, 1512,<br>1617, 1722, 1737
**20**	6S	isLabeled, 1017, 1077, 1212,<br>1452, 1467, 1482,<br>1527, 1587, 1737
**18,19,21**	6S	1017, 1122, 1212, 1227,<br>1527, 1737
**18,20,21,22**	6S	1092, 1152, 1182, 1212,<br>1302, 1332, 1467,<br>1482, 1512, 1527,<br>1587, 1737, 1782
**17,18,19,21**	6S	1212, 1287, 1452, 1467,<br>1482
**19,21,22**	6S	1017, 1197, 1212, 1227,<br>1287, 1377, 1452,<br>1467, 1482, 1497,<br>1512, 1737
**19,20**	6S	1122, 1182, 1212, 1227,<br>1287, 1407, 1467,<br>1482, 1527, 1632,<br>1647, 1707, 1737
**19,20,22**	6S	isLabeled, 1032, 1047, 1122,<br>1197, 1467
**18,19,21,22**	6S	1122, 1152, 1212, 1227,<br>1467, 1512, 1707,<br>1722, 1737
**19,20,21,22**	6S	isLabeled, 1047, 1092, 1152,<br>1212, 1227, 1377,<br>1467, 1632, 1677,<br>1722, 1737, 1752,<br>1767

**18,19,22**	6S	Charge, isLabeled, 1197, 1212, 1287, 1392, 1452, 1617
**17,19**	6S	isLabeled, 1212, 1227, 1287, 1467, 1482, 1737, 1752
**17,18**	6S	1212, 1287, 1467, 1572, 1737
**17,18,19,22**	6S	1152, 1212, 1287, 1377, 1392, 1467, 1482, 1707
**18,22**	6S	1047, 1212, 1467, 1482, 1617, 1662, 1737
**18,19**	6S	Charge, 1107, 1197, 1212, 1302, 1332, 1467, 1482, 1512, 1632, 1737
**17,19,20,22**	6S	Charge, 1047, 1137, 1182, 1197, 1212, 1227, 1257, 1347, 1482, 1527, 1647
**17,22**	6S	1107, 1212, 1332, 1392, 1467, 1737
**17,21**	6S	1182, 1392, 1467, 1512, 1737
**17**	6S	1287, 1467, 1497, 1722
**17,18,19**	6S	1212, 1287, 1347, 1467, 1737
**18,20,22**	6S	1212, 1272, 1287, 1467, 1752
**17,18,19,20,22**	6S	Charge, 1212, 1467, 1482, 1587, 1722

References

(1) Lettow, M.; Greis, K.; Grabarics, M.; Horlebein, J.; Miller, R. L.; Meijer, G.; von Helden, G.; Pagel, K. Chondroitin Sulfate Disaccharides in the Gas Phase: Differentiation and Conformational Constraints. *J. Phys. Chem. A* **2021**, *125*, 4373–4379, DOI: 10.1021/acs.jpca.1c02463.

(2) Lettow, M.; Grabarics, M.; Greis, K.; Mucha, E.; Thomas, D. A.; Chopra, P.; Boons, G.-J.; Karlsson, R.; Turnbull, J. E.; Meijer, G., et al. Cryogenic Infrared Spectroscopy Reveals Structural Modularity in the Vibrational Fingerprints of Heparan Sulfate Diastereomers. *Anal. Chem.* **2020**, *92*, 10228–10232, DOI: 10.1021/acs.analchem.0c02048.

(3) Géron, A., *Hands-On Machine Learning with Scikit-Learn, Keras, and TensorFlow: Concepts, Tools, and Techniques to Build Intelligent Systems*; O'Reilly Media, Inc.: 2019.

(4) Pedregosa, F.; Varoquaux, G.; Gramfort, A.; Michel, V.; Thirion, B.; Grisel, O.; Blondel, M.; Prettenhofer, P.; Weiss, R.; Dubourg, V., et al. Scikit-learn: Machine Learning in Python. *J. Mach. Learn. Res.* **2011**, *12*, 2825–2830.

(5) Hastie, T.; Tibshirani, R.; Friedman, J. H.; Friedman, J. H., *The Elements of Statistical Learning: Data Mining, Inference, and Prediction*; Springer: 2009; Vol. 2.

(6) sklearn-genetic-opt, accessed: 02/05/2022, 2022, https://sklearn-genetic-opt.readthedocs.io.

(7) Eiben, A. E.; Smith, J. E., *Introduction to Evolutionary Computing*; Springer: 2015.
